# Live Imaging and Gene Expression Analysis in Zebrafish Identifies a Link between Neutrophils and Epithelial to Mesenchymal Transition

**DOI:** 10.1371/journal.pone.0112183

**Published:** 2014-11-05

**Authors:** Christina M. Freisinger, Anna Huttenlocher

**Affiliations:** Departments of Pediatrics and Medical Microbiology and Immunology, University of Wisconsin-Madison, Madison, Wisconsin, United States of America; Ghent University, Belgium

## Abstract

Chronic inflammation is associated with epithelial to mesenchymal transition (EMT) and cancer progression however the relationship between inflammation and EMT remains unclear. Here, we have exploited zebrafish to visualize and quantify the earliest events during epithelial cell transformation induced by oncogenic HRas^V12^. Live imaging revealed that expression of HRas^V12^ in the epidermis results in EMT and chronic neutrophil and macrophage infiltration. We have developed an *in vivo* system to probe and quantify gene expression changes specifically in transformed cells from chimeric zebrafish expressing oncogenic HRas^V12^ using translating ribosomal affinity purification (TRAP). We found that the expression of genes associated with EMT, including *slug*, *vimentin* and *mmp9*, are enriched in HRas^V12^ transformed epithelial cells and that this enrichment requires the presence of neutrophils. An early signal induced by HRas^V12^ in epithelial cells is the expression of *il-8* (*cxcl8*) and we found that the chemokine receptor, Cxcr2, mediates neutrophil but not macrophage recruitment to the transformed cells. Surprisingly, we also found a cell autonomous role for Cxcr2 signaling in transformed cells for both neutrophil recruitment and EMT related gene expression associated with Ras transformation. Taken together, these findings implicate both autocrine and paracrine signaling through Cxcr2 in the regulation of inflammation and gene expression in transformed epithelial cells.

## Introduction

Epithelial to mesenchymal transition (EMT) is essential for normal embryonic development, but also occurs during wound healing and the invasion of transformed cells during cancer progression [1], [2]. One main difference between the activation of programmed EMT during early development and EMT associated with pathology is the presence of inflammation [3]. Chronic inflammation associated with diseases like Rheumatoid Arthritis, Crohns disease, Chronic obstructive pulmonary disease (COPD) and pancreatitis is known to increase cancer risk [4], [5] and neutrophil depletion has been shown to reduce tumor burden in a mouse model of lung cancer [6]. The connection between inflammation and cancer risk is further supported by studies that suggest that treatment with anti-inflammatory agents can decrease the incidence of some cancers [7]–[9]. Although chronic inflammation has been linked to EMT and cancer invasion [10]–[12], it is not clear how innate immune inflammation is associated with EMT and participates in cancer progression.

Understanding how transformed cells induce chronic innate immune inflammation in tissues and if this inflammation plays a role in EMT progression has been limited due to the difficulty visualizing the early tissue microenvironment around small clusters of transformed epithelial cells in live animals. Zebrafish is therefore an ideal model system to study these questions since zebrafish larvae are transparent allowing for real time imaging of EMT in live animals. Indeed, zebrafish have emerged as a powerful model system to study the pathogenesis of cancer since many of the signaling pathways and mechanisms are conserved [13], [14]. In fact, recent progress in zebrafish has identified signaling pathways that mediate leukocyte recruitment to wounds [15]–[17], infection [18] and transformed melanocytes [19]. Additionally, chronic inflammation of the epidermis in zebrafish has been associated with the activation of EMT programs and developmental defects in epidermal homeostasis, further supporting the use of zebrafish to study the relationship between inflammation and EMT [10], [20].

Chemokine-dependent signaling in immune cells is an important mechanism that mediates leukocyte recruitment to bacterial infections and wounds. A large body of work has explored the role of the chemokine (C-X-C motif) receptor 2 (CXCR2) during leukocyte recruitment to sites of inflammation [6], [15]–[19], [21]–[23]. Previous studies, utilizing the zebrafish model system, have shown that Cxcr2 receptor signaling axis is involved in both long-range systemic neutrophil mobilization from hematopoietic tissue as well as local recruitment to infection or purified Cxcl8 (IL-8, a potent Cxcr2 ligand) [17], [18]. Previous studies have also implicated CXCR2 signaling in tumor progression [21], [22], [24]–[29]. CXCR2 expression is increased in some tumor types [26], [30]–[32] and pharmacological inhibition of CXCR1 and CXCR2 inhibits neutrophil recruitment into A547 lung tumor spheroids resulting in slower tumor growth [33]. Mouse models have also been instrumental in demonstrating that CXCR2 signaling is involved in the recruitment of myeloid-derived suppressor cells into the tumor microenvironment. For instance, expression of human CXCL8 in mice results in increased mobilization of immature myeloid cells, which exacerbates inflammation and accelerates colon carcinogenesis [34]. Tumor trafficking of myeloid-derived suppressor cells is inhibited by CXCR2 deficiency in a mouse model of rhabdomyosarcoma [35] and data from a mouse model of colitis-associated cancer suggests that CXCR2 is required for recruitment of myeloid-derived suppressor cells [24], [36]. Moreover, activation of CXCR2 on Ras-transformed keratinocytes contributes to tumor progression in a mouse model of skin cancer [23]. These findings support a role for CXCR2 signaling in inflammation and cancer progression; however the connection between CXCR2-mediated neutrophil recruitment and EMT remains unclear.

In this study we have exploited advances in real time imaging and analysis of tissue-specific gene expression in zebrafish to interrogate the role of Cxcr2 and neutrophil recruitment in HRas^V12^-induced EMT. We found that signaling through Cxcr2 is required for neutrophil, but not macrophage, recruitment to transformed epithelial cells. We further show that both Cxcr2 in transformed cells and the presence of neutrophils, but not macrophages, in the microenvironment are required for expression of EMT-related genes. These findings establish an essential role for Cxcr2 in regulating chronic neutrophil inflammation and EMT-related gene expression via both autocrine and paracrine mechanisms.

## Results

### HRas^V12^ expression in epithelial cells induces EMT and inflammation

**Figure 1:**
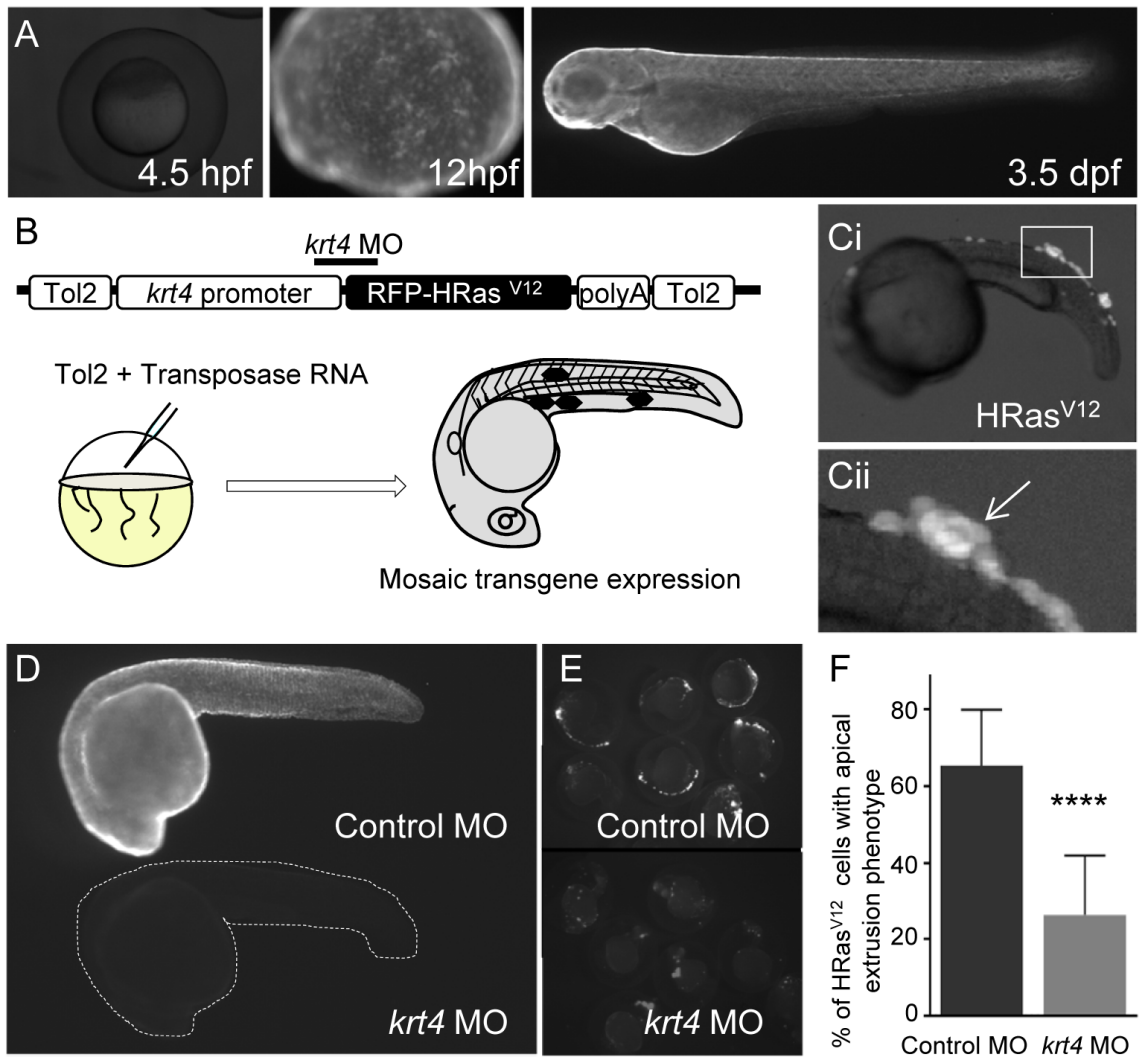
Early HRas^V12^ expression in epithelial cells induces cell extrusion. (A) Lateral fluorescent images of transgenic *krt4*-GFP embryos at 4.5 hpf, 12 hpf and 3 dpf. (B) Schematic of the Tol2 flanked:*krt4*:RFP-HRas^V12^ construct+transposase one-cell stage injection resulting in mosaic RFP-HRas expression by 24 hpf (black hexagons). (C) (i) Lateral fluorescent image of a 24 hpf live H-Ras^V12^ expressing embryo. (ii) High-magnification view of the inset in (i) indicating the apical cell extrusion phenotype (white arrow). (D) Fluorescent images of live 24 hpf *krt4*:GFP transgenic embryos injected with either control MO (Top) or Krt4 MO (bottom dashed outline) at 24 hpf illustrating that the *krt4* MO reduces transgene levels in a stable transgenic line. (E) Fluorescent images of live 24 hpf embryos transiently expressing HRas^V12^ injected with either control MO or *krt4* MO illustrating that the *krt4* MO reduces transgene levels in embryos with mosaic transgene expression. (F) Quantification of cell extrusion (one representative graph shown n = 3) of HRas^V12^ expressing cells from control MO and Krt4 MO injected embryos shows a significant decrease in the cell extrusion in embryos injected with the Krt4 MO. **** = p<.0001.

Tissue damage or oncogenic signals induce EMT [37]. To visualize EMT in zebrafish, we used the *krt4* promoter to drive gene expression in epidermal cells (*krt4*; previously termed *krt8*) [38]. We found that the *krt4* promoter drives GFP expression in the developing epidermis during early gastrulation and by 12 hours post fertilization (hpf) uniform expression is observed throughout the epidermis (Figure 1A). To express HRas^V12^ in the zebrafish epidermis, RFP-HRas^V12^ was cloned into a Tol2 containing plasmid and co-injected with transposase RNA into one-cell stage embryos (Figure 1B). Active Ras signaling promotes cell transformation [39]–[43] and has been shown to drive chemokine and cytokine expression [44]–[46]. Early expression of HRas^V12^ using the *krt*4 promoter induced cell extrusion in developing embryos (Figure 1C), as has previously been reported for apoptotic epithelial cells in zebrafish [47] or in response to activating Src [48]. To reduce early transgene expression, we designed an antisense morpholino oligonucleotide (MO) targeting the 3′ end of the *krt4* promoter 25 bases directly 5′ to the AUG translational start site of RFP-HRas^V12^ (Figure 1B). Microinjection of the *krt4* MO inhibited GFP expression in *krt4*:GFP transgenic larvae (Figure 1D) and reduced transgene expression in 24 hpf embryos injected with Tol2 flanked:*krt4*: RFP-HRas^V12^ (Figure 1E). By reducing early HRas^V12^ transgene expression, we were able to significantly reduce the early apical extrusion of HRas^V12^ expressing cells (Figure 1F).

**Figure 2:**
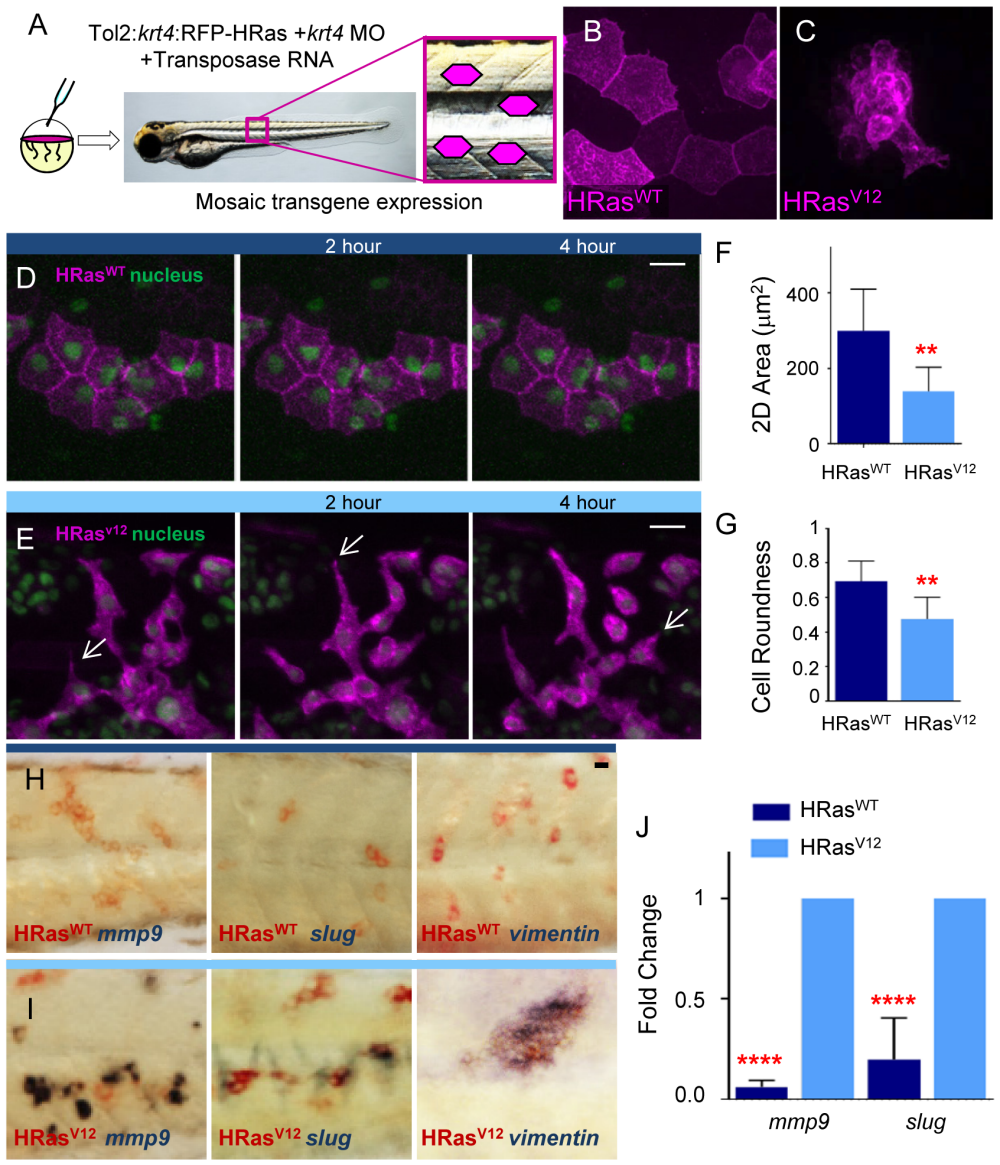
HRas^V12^ expression in epithelial cells induces cell shape and genetic changes associated with EMT *in vivo*. (A) Schematic of Tol2 flanked:*krt4*:RFP-HRas+transposase one-cell stage injection resulting in mosaic expression. (B–C) Fluorescent Z stack projections of HRas^WT^ and HRas^V12^ expressing epithelial cells (magenta) in the trunk region of 3.5 dpf larvae (illustrated in A). (D–E) Lateral fluorescent images, from live imaging, of 3.5 dpf embryos co-expressing GFP-H2B to label the nuclei and either HRas^WT^ (D) or HRas^V12^ (E) at 0, 2 hr and 4 hr time points. Arrows in E indicate cell extensions. (F) Quantification of the 2D area of H-Ras^WT^ and H-Ras^V12^ expressing cells shows a significant decrease in the 2D area of HRas^V12^ expressing cells compared to controls. (G) Quantification of the cell roundness of HRas^WT^ and HRas^V12^ expressing cells shows a significant decrease in the cell roundness of HRas^V12^ expressing cells compared to controls. (H–I) Double label WMISH with HRas^WT^ (H) and HRas^V12^ (I) transcript labeled in red and *mmp9*, *slug*, and *vimentin* transcript label in blue illustrating that *mmp9*, *slug*, and *vimentin* expression are induced in RFP-HRas^V12^ compared to control RFP-HRas^WT^ expressing larvae. (J) Quantitative RT-PCR (one representative graph shown n = 5) indicates a statistically significant increase in *mmp9* and *slug* transcripts in HRas^V12^ transformed cells compared to control HRas^WT^ expressing cells. hr = hour, dpf = days post fertilization, ** = p<.005, **** = p<.0001 scale bars = 20 microns.

High resolution confocal imaging revealed that chimeric embryos expressing wild type HRas^WT^ had membrane localization of the transgene and displayed a cuboidal morphology typical of epithelial cells at 3.5 days post fertilization (dpf) (Figure 2B). Cells expressing constitutively active HRas^V12^ also had membrane localization of the transgene but displayed altered cell morphology (Figure 2C). Live imaging, of chimeric 3.5 dpf embryos, revealed that HRas^WT^ cells maintained their shape over a four hour time period (Figure 1D, Movie S1) while, HRas^V12^ cells displayed an abnormal morphology with dynamic protrusions (Figure 2E, Movie S2), quantified by reduced 2D cell area and roundness (Figure 2F–G).

To determine if HRas^V12^ expression in epithelial cells resulted in changes in gene expression consistent with EMT, we investigated the expression of a transcriptional activator of EMT, Slug (also known as Snail2) which has been identified as a driving factor of EMT in keratinocytes during wound healing [37] and is increased during cancer progression [49]. We also tested expression of a matrix metalloproteinase (Mmp9) that has been linked to EMT, and a type III intermediate filament protein (Vimentin) that is expressed in mesenchymal cells and has been previously shown to be a reliable marker of EMT [50]–[52]. Double label Whole Mount *In Situ* Hybridization (WMISH) revealed that the EMT associated genes *mmp9*, *slug* and *vimentin* were enriched in HRas^V12^ transformed epithelial cells, compared to control HRas^WT^ expressing cells (Figure 2H–I). To better quantify these changes in gene expression we used translating ribosome affinity purification (TRAP) [53] to isolate RNA specifically from the transformed epithelial cells followed by Quantitative Reverse Transcriptase Polymerase Chain Reaction (qRT-PCR). We found that transforming HRas^V12^ induced EMT gene expression specifically in the transformed epithelial cells, including increased *slug* and *mmp9*, compared to HRas^WT^ control at 3.5 dpf (Figure 2J).

**Figure 3:**
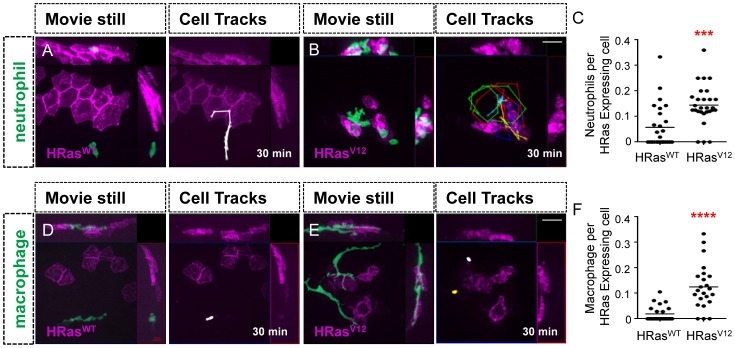
Leukocytes are recruited to HRas^V12^ expressing epithelial cells. (A–B) Analysis of time-lapse movies of control HRas^WT^ expressing epithelial cells (A and D) and HRas^V12^ expressing epithelial cells (B and E) in 3.5 dpf transgenic *mpx*:GFP (green neutrophils) larvae (A–B) and 3.5 dpf transgenic *mpeg*:Dendra (green macrophage) larvae (D–E). For cell tracks leukocyte migration was tracked every 2 minutes for 30 minutes. (C) Quantification of A–B (as a ratio of neutrophils per transformed cell) confirms a statistically significant increase in neutrophil recruitment to HRas^V12^ expressing cells compared to HRas^WT^ expressing cells. (F) Quantification of D–E (as a ratio of macrophages per transformed cell) confirms a statistically significant increase in macrophage recruitment to HRas^V12^ expressing cells when compared to macrophages recruited to HRas^WT^ expressing cells. dpf = days post fertilization, scale bar = 20 microns, *** = p<.001 **** = p<.0001.

To characterize the host innate immune responses to transformed epithelial cells, we used the transgenic zebrafish lines Tg(*mpx*:GFP) and Tg(*mpeg*:Dendra) to visualize neutrophils and macrophages respectively *in vivo* [20], [54]–[56]. In zebrafish larvae, neutrophils are generally localized to the caudal hematopoietic tissue (CHT) [57], which has a hematopoietic function similar to the fetal liver in mammals [58]. Live high resolution spinning disk confocal microscopy revealed that neutrophils (Figure 3B, Movie S4) and macrophages (Figure 3E, Movie S6) are recruited to HRas^V12^ but not control HRas^WT^ expressing epithelial cells (Figure 3A and D, Movie S3 and S5). Quantification of leukocytes as a ratio of transformed cell number revealed a significant increase in neutrophils (Figure 3C) and macrophages (Figure 3F) per HRas^V12^ transformed cell compared to control HRas^WT^ cells. These findings indicate that chimeric HRas^V12^ expression in the zebrafish epidermis is sufficient to stimulate neutrophil and macrophage recruitment, similar to the effects reported with oncogene-transformed melanoblasts in zebrafish [19].

### Neutrophils, but not macrophages, are necessary for *mmp9* and *slug* expression in transformed epithelial cells

**Figure 4:**
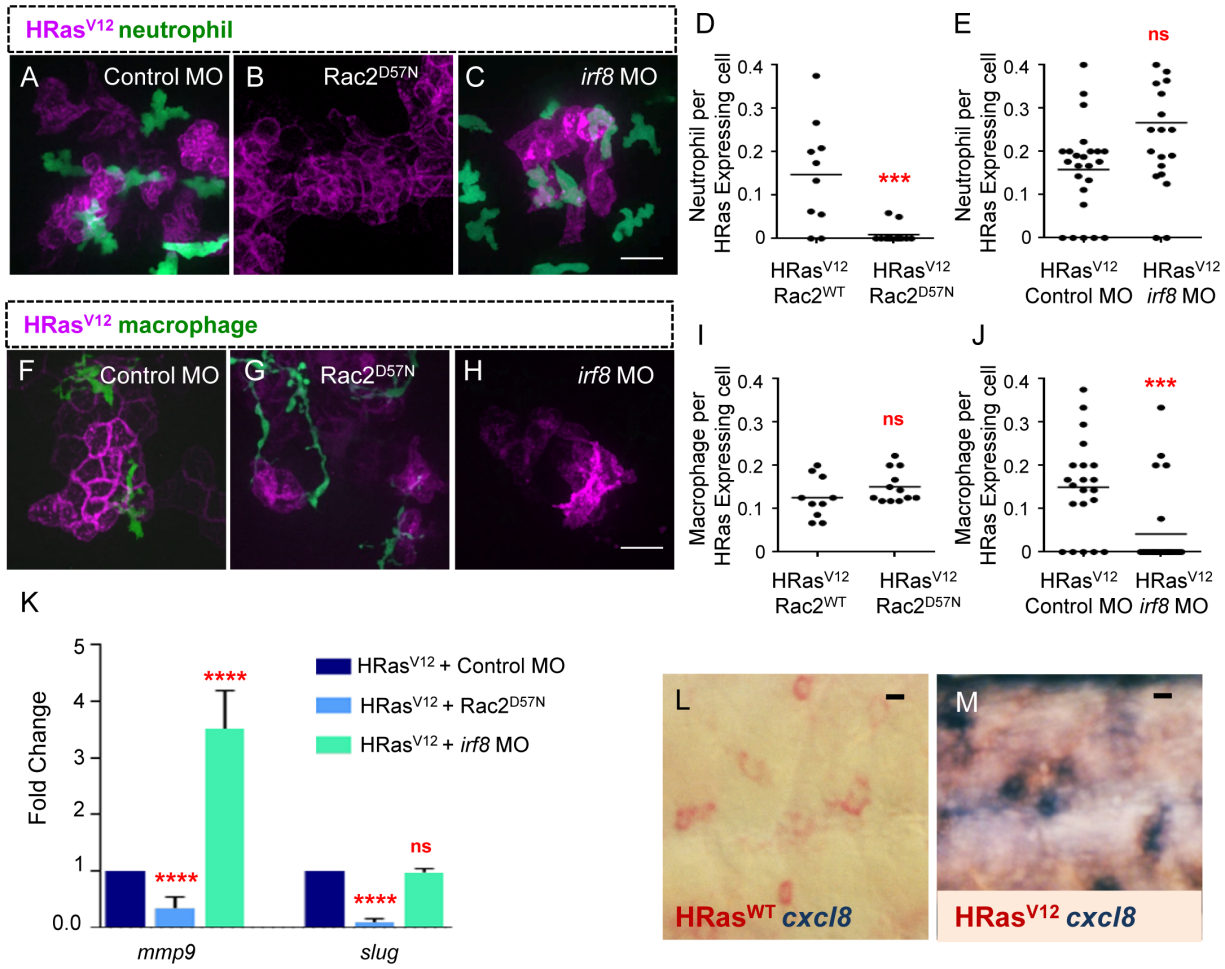
Neutrophils, but not macrophages, mediate EMT related gene expression in HRas^V12^ expressing epithelial cells. (A–C) Fluorescent Z stack projections of live 3.5 dpf transgenic *mpx*:GFP (green neutrophils) control MO injected (A), Rac2^D57N^ (B) and *irf8* MO (C) larvae expressing HRas^V12^. (D–E) Quantification of neutrophil recruitment (as a ratio of neutrophils per transformed cell) shows a significant decrease in neutrophil recruitment to HRas^V12^ expressing cells in Rac^D57N^ embryos when compared to controls (D), no significant change was observed in neutrophil recruitment in *irf8* morphant larvae compared to control (E). (F–H) Fluorescent Z stack projections of live 3.5 dpf of transgenic *mpeg*:Dendra (green macrophages) control MO injected (F), Rac2^D57N^ (G) and *irf8* MO (H) larvae expressing HRas^V12^. (I–J) Quantification of macrophage recruitment (as a ratio of macrophages per transformed cell) shows a significant decrease in macrophage recruitment to HRas^V12^ expressing cells in irf8 morphants compared to controls (D). No significant change was observed in macrophage recruitment in Rac2^D57N^ larvae compared to control (E). (K) Quantitative RT-PCR (one representative graph shown n = 4) indicates a statistically significant decrease in *mmp9* and *slug* transcripts in transformed cells from Rac2^D57N^ larvae compared to control MO injected larvae while no significant decrease was seen in *mmp9* and *slug* transcripts in transformed cells from *irf8* Mo injected larvae compared to controls. (L–M) Double label WMISH with HRas^WT^ (A) and HRas^V12^ (B) transcript labeled in red and *cxcl8* transcript label in blue. *cxcl8* expression is induced in HRas^V12^ expressing larvae compared to control HRas^WT^ expressing larvae. *** = P<.001, **** = P<.0001, ns = not significant. Scale bar = 20 microns.

To determine if there is a cell autonomous role for neutrophils in regulating EMT, we characterized EMT related gene expression in larvae with impaired neutrophil function. We took advantage of a zebrafish model of primary immunodeficiency (zf307Tg, Tg(mpx:mCherry,rac2^D57N^)), where neutrophil recruitment to tissue damage is impaired. In this model, expression of the human inhibitory Rac2^D57N^ mutation in neutrophils results in reduced neutrophil migration and recruitment to wounds and infection [57]. We found a significant decrease in neutrophil recruitment to HRas^V12^ expressing cells in Rac2^D57N^ larvae compared to control (Figure 4B and D). To ensure that macrophages were still recruited to transformed cells in the absence of neutrophil recruitment we quantified macrophage numbers at transformed cells in Rac2^D57N^ larvae and found that macrophage recruitment was not affected (Figure 4G and I). Surprisingly, we found that EMT associated gene expression is impaired in neutrophil-deficient larvae (Figure 4K) indicating that neutrophils are necessary for the expression of EMT associated genes in transformed epithelial cells.

To determine if there is a cell autonomous role for macrophages in regulating EMT associated gene expression, we utilized a previously published MO targeting interferon regulatory factor 8 (*Irf8*), which is essential for directing macrophage but not neutrophil differentiation [59]. We found a significant decrease in macrophage recruitment to HRas^V12^ expressing cells in *irf8* morphants compared to control (Figure 4 H and J). To determine if neutrophils were still recruited to transformed cells in *irf8* morphants we quantified neutrophil numbers at transformed cells and found that neutrophil recruitment was not affected (Figure 4C and E). Moreover, *mmp9* and *slug* transcripts were not reduced in *irf8* morphants compared to control (Figure 4K), suggesting that macrophages do not induce EMT gene expression in transformed cells. Interestingly, *mmp9* expression was increased in *irf8* morphants, likely due to the increase in total numbers of neutrophils in *irf8* morphants. Taken together, these findings suggest that neutrophils but not macrophages influence EMT associated gene expression in transformed epithelial cells *in vivo*.

### Cxcr2 is required for neutrophil recruitment to transformed cells

Previous work has predominantly focused on the role of macrophages in cancer progression and few studies have characterized the role of neutrophils within the tumor microenvironment [60]–[65]. Our findings suggest that neutrophils play a critical role in influencing gene expression in transformed epithelial cells. To identify the signals that mediate neutrophil recruitment to transformed epithelial cells, we targeted specific pathways that have been implicated in neutrophil wound attraction, including Cxcl8 signaling [66]. Indeed, human liver epithelial cells from hepatocellular carcinoma have been shown to produce the CXC chemokine CXCL8 and promote neutrophil infiltration [67]. To determine if Cxcl8 is up-regulated by epithelial cell transformation we performed double label WMISH and found an increase in Cxcl8 in regions with HRas^V12^ expressing cells (Figure 4M), compared to HRas^WT^ control (Figure 4L).

**Figure 5:**
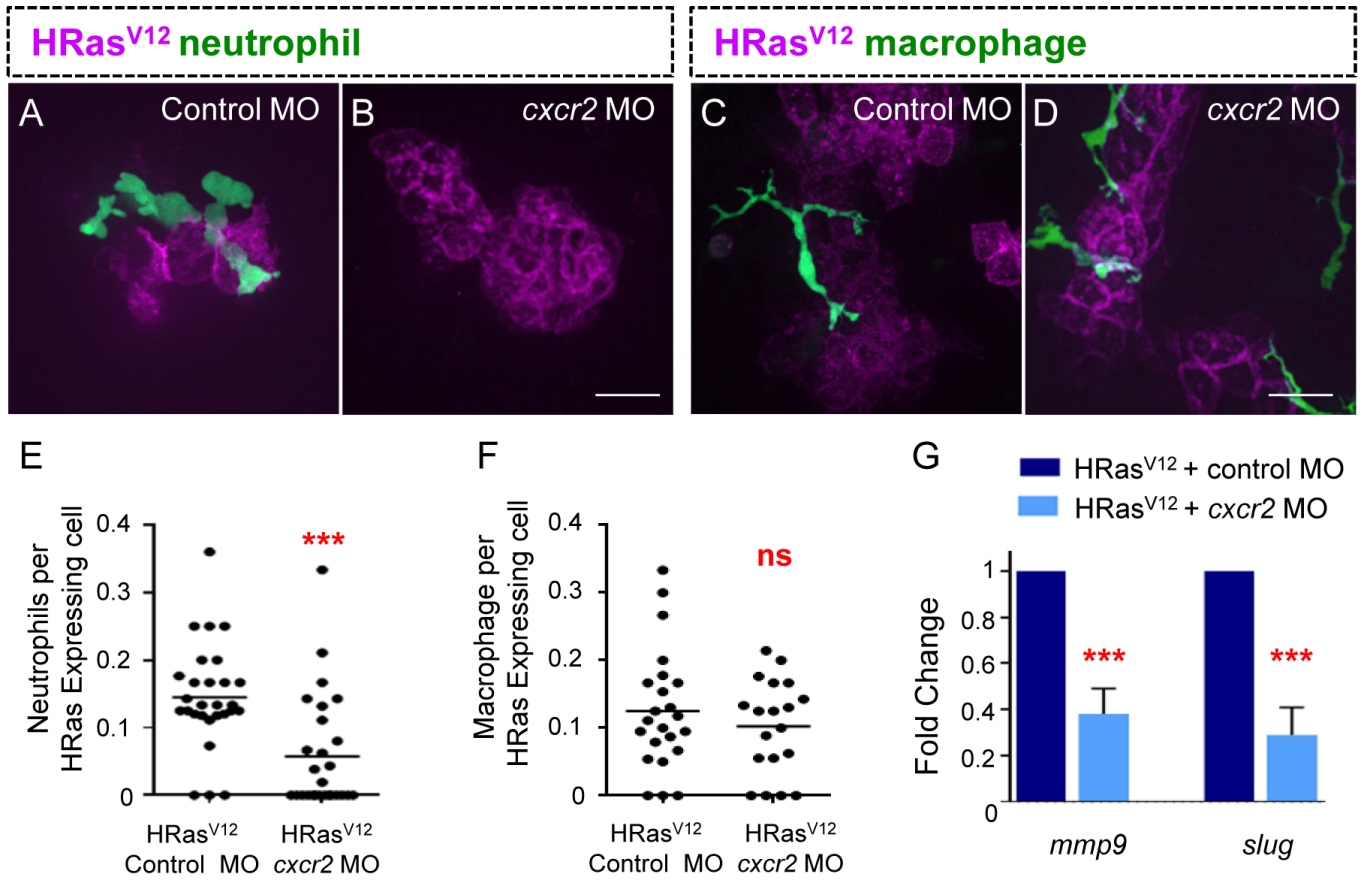
Cxcr2 is required for EMT related gene expression in HRas^V12^ expressing epithelial cells. (A–B) Fluorescent Z stack projections of live 3.5 dpf transgenic *mpx*:GFP (green neutrophils) control MO injected (A) and *cxcr2* morphant (B). (C–D) Fluorescent Z stack projections of live 3.5 dpf transgenic *mpeg*:Dendra (green macrophages), control MO injected (C) and *cxcr2* morphant (D). (E) Quantification of A–B (as a ratio of neutrophils per transformed cell) reveals a significant decrease in neutrophil recruitment to HRas^V12^ expressing cells in *cxcr2* MO injected larvae compared to control. (F) Quantification of C–D (as a ratio of macrophages per transformed cell) shows that macrophage numbers at HRas^V12^ expressing cells in *cxcr2* MO injected larvae is similar to macrophage numbers at HRas^V12^ expressing cells in control larvae. (G) Quantitative RT-PCR (one representative graph shown n = 4) indicates a statistically significant decrease in *mmp9* and *slug* transcripts in transformed cells from cxcr2 MO injected larvae when compared to control MO injected larvae. *** = P<.001, ns = not significant. Scale bar = 20 microns.

We recently reported that Cxcr2, but not Cxcr1, is necessary for neutrophil recruitment to exogenous purified Cxcl8, suggesting that Cxcr2 may mediate neutrophil recruitment to transformed epithelial cells that produce Cxcl8 [18]. To determine if Cxcr2 mediates neutrophil recruitment we depleted *cxcr2* using a previously published MO [18]. We found that depletion of *cxcr2* resulted in a significant decrease in neutrophil recruitment to HRas^V12^ expressing cells, quantified as a ratio of neutrophils per transformed cell (Figure 5B and E). To determine if Cxcr2 also mediates macrophage recruitment we quantified macrophage infiltration, and found that, *cxcr2* depletion did not have a significant impact on macrophage recruitment (Figure 5D and F), suggesting that Cxcr2 mediates neutrophil but not macrophage recruitment to transformed epithelial cells.

To determine if Cxcr2 mediates the invasive progression of transformed cells we characterized the effect of *cxcr2* depletion on the HRas^V12^-induced expression of *mmp9* and *slug*. We found that depletion of *cxcr2* blocked the HRAS^V12^-induced expression of EMT related genes (Figure 5G). It is important to note that, although neutrophil recruitment required Cxcr2, the early morphological changes induced by HRas^V12^ were not affected by *cxcr2* depletion. These findings indicate the Cxcr2 is necessary for HRAS^V12^-induced expression of EMT associated genes.

### Cxcr2 signaling in transformed epithelial cells is necessary for neutrophil recruitment and HRas^V12^ induced expression of *vimentin*, *mmp9* and *slug* in epithelial cells

**Figure 6:**
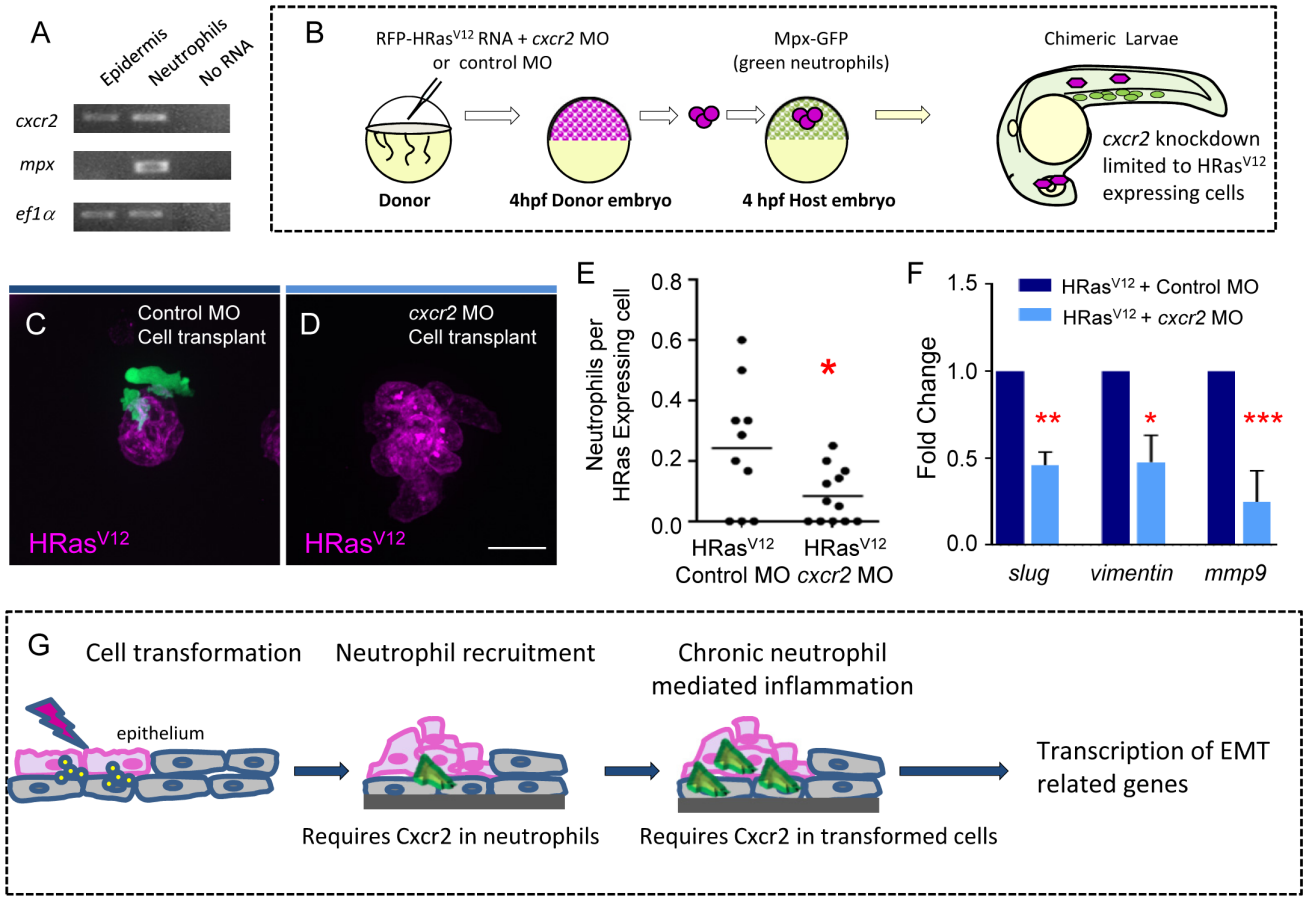
Cxcr2 signaling in HRas^V12^ transformed epithelial cells is required for neutrophil recruitment and EMT related gene expression. (A) For analysis of tissue specific Cxcr2 expression TRAP was performed on 3.5 dpf transgenic krt4-EGFP-L10a and mpx-EGFP-L10a larvae and one-step RT-PCR was performed. *cxcr2* expression is observed in the epidermis and in neutrophils. *mpx* expression is only observed in neutrophils supporting that there is not neutrophil contamination in the epidermal samples. (B) Schematic diagram to illustrate the cell transplantation used to generate chimeric HRas^V12^ expressing larvae in which the transformed cells express either control MO or Cxcr2 MO. (C–D) Fluorescent Z stack projections of live 3.5 dpf of transgenic *mpx*:GFP (green neutrophils) larvae with control MO in the HRas^V12^ expressing cells (C) or with *cxcr2* MO within the HRas^V12^ expressing cells (D). (E) Quantification of C–D (as a ratio of neutrophils per transformed cell) shows a statistically significant decrease in neutrophil recruitment to HRas^V12^ expressing cells that have *cxcr2* MO compared to HRas^V12^ expressing cells that have control MO. (F) Quantitative RT-PCR (one representative graph shown n = 3) indicates a statistically significant decrease in *slug*, vimentin and *mmp9* transcripts in HRas^V12^ expressing cells that have *cxcr2* MO compared to HRas^V12^ expressing cells with control MO. (G) Schematic illustrating the requirement for Cxcr2 in neutrophils for initial neutrophil recruitment to transformed cells as well as a cell autonomous function of Cxcr2 in transformed cells to mediate changes associated with EMT. * = P<.05, ** = P<.01, *** = P<.001. Scale bar = 20 microns.

Previous studies have shown that Cxcr2 expression is necessary for neutrophil recruitment to exogenous Cxcl8, indicating that neutrophil Cxcr2 mediates chemotaxis to Cxcl8 *in vivo*. It is possible that Cxcr2 mediates EMT gene expression in transformed epithelial cells indirectly through its effects on neutrophil recruitment. However, Cxcr2 is also expressed in tumor cells, suggesting that Cxcr2 may play cell autonomous roles in epithelial cells. Indeed, we found that zebrafish epithelial cells express *cxcr2* (Figure 6A), suggesting that Cxcr2 in epithelial cells may affect gene expression changes induced by cell transformation independent of the effects of Cxcr2 in neutrophils. To determine if there is a cell autonomous role for Cxcr2 in transformed epithelial cells we utilized a cell transplantation strategy to deplete Cxcr2 specifically in transformed cells without altering Cxcr2 signaling in neutrophils (Figure 6B). Cells from embryos expressing RFP-HRas^V12^ in *cxcr2* morphants or control morphants were transplanted into Tg(mpx:GFP) embryos. Interestingly, we found a significant decrease in neutrophil recruitment toward Cxcr2-deficient HRas^V12^ expressing cells even though the neutrophils expressed Cxcr2 (Figure 6D and E), suggesting that Cxcr2 signaling in transformed cells is necessary for neutrophil recruitment. To determine if Cxcr2-deficient epithelial cells that express HRas^V12^ induce EMT associated genes, we tested the expression of *vimentin*, *mmp9* and *slug* in the Cxcr2-deficient and control transformed epithelial cells. Surprisingly, expression of *vimentin*, *slug* and *mmp9* were reduced in Cxcr2-deficient transformed epithelial cells compared to control (Figure 6F); indicating a cell autonomous role for Cxcr2 signaling in epithelial cells in the induction of EMT related gene expression.

## Discussion

Here, we uncovered roles for Cxcr2 and neutrophils in oncogene induced EMT gene expression in epithelial cells in zebrafish. By exploiting the transparency of zebrafish we showed that expression of oncogenic HRas^V12^ induced EMT and invasive growth. Interestingly, early expression of HRas^V12^ induced cell extrusion, suggesting that depending on the developmental stage of the epithelium the fate of the transformed cell may be either extrusion or EMT. This provides a powerful model system to understand how early EMT is regulated within a live host, and how different triggers for EMT during development, cell transformation or wounding induce distinct EMT programs.

Previous studies have identified roles for both macrophages [60]–[65] and neutrophils [6], [33], [68] during cancer progression. For example, neutrophil activation is associated with the progression of head and neck cancers [68] supporting the idea that neutrophils influence tumor progression. CXCR2 has a known role in the recruitment of neutrophils to cancer cells. Of interest CXCR2 expressing ovarian cancers are aggressive with poor outcomes [69]. However, the mechanisms of these effects remain poorly understood.

Here we show directly that depletion of Cxcr2 in transformed cells results in decreased neutrophil recruitment and impairs the early expression of EMT genes in transformed epithelial cells. Our findings reveal a pro-inflammatory role for Cxcr2 in Ras transformed keratinocytes and provides direct evidence that neutrophils impact the progression of transformed cells in a live animal. It is likely that chronic neutrophil infiltration results in increased amounts of neutrophil derived elastase which has been previously shown to support the progression of pancreatic tumor cells [70] and lung cancer in mice [6]. To test this possibility we exposed zebrafish larvae transiently expressing HRas^V12^ in the epidermis to a specific inhibitor of human neutrophil elastase (Sivelestat) from 1–3.5 dpf, however we did not observe any difference in either neutrophil recruitment or the transcription of EMT related genes. It is possible that Sivelestat may not be effective in inhibiting zebrafish neutrophil elastase and future experiments are needed to determine the role of neutrophil derived elastase in the transcription of EMT related genes. Additionally, it is also possible that a positive feedback loop between neutrophil derived Cxcl8 and Cxcr2 signaling in the transformed cells may contribute to these changes. This is especially interesting since recent evidence suggests that CXCL8 is associated with progression of some cancers [45].

The zebrafish model is particularly powerful since the transparency allows for the real time visualization of the earliest events that occur after an oncogene turns on. We found that early after HRas^V12^ was first expressed in epithelial cells both neutrophils and macrophages are recruited to small foci of transformed cells. A previous report showed that transformed melanoblasts in zebrafish also induces early leukocyte recruitment [19]. The previous study showed that hydrogen peroxide generated by the transformed cells mediates leukocyte recruitment. We found that reactive oxygen species inhibition with DPI did not impair neutrophil recruitment to the transformed epithelial cells, but this may have been due to the short time of inhibitor treatment as longer treatments resulted in lethality. It is also possible that different signals mediate neutrophil recruitment to transformed melanocytes versus epithelial cells. Our findings support an essential role for Cxcr2 signaling in chronic tumor associated inflammation.

In summary, here we report a new *in vivo* model of epithelial cell transformation that is amenable to live imaging and probing the microenvironment. We have identified a pathway that specifically mediates neutrophil but not macrophage recruitment to transformed epithelial cells. We have also provided evidence that Cxcr2 and neutrophils are both necessary for HRas^V12^-induced changes in gene expression in epithelial cells (Figure 6G). This study highlights a novel cell autonomous role for Cxcr2 signaling in transformed cells in both influencing neutrophil infiltration and EMT-related gene expression, suggesting that both autocrine and paracrine signaling contribute to Cxcr2 effects on EMT. Future studies will be necessary to identify the specific neutrophil factors that influence gene expression induced by oncogenic HRas. It is possible that neutrophils regulate gene expression in transformed epithelial cells at least in part through the release of Cxcl8 that signals to epithelial cell Cxcr2 in a positive feedback loop. The zebrafish model is poised to contribute to a better understanding of the trophic factors involved in the reactivation of EMT programs *in vivo*, which will likely aid in identifying novel therapeutic targets that modulate EMT with implications to both wound and cancer biology.

## Materials and Methods

### Zebrafish maintenance and general procedures

University of Wisconsin - Madison (A3368-01) Institutional review board specifically approved this study. Adult AB fish, including Transgenic (Tg) zebrafish lines Tg(*mpx*:GFP), Tg(*mpeg*:Dendra), Tg(*mpx*: EGFP-L10a [53]), Tg(*krt4*: EGFP-L10a [53]) and Tg(mpx:mCherry,rac2^D57N^, zf307Tg [57]) were maintained at 28°C in a 14-hour light/10-hour dark cycle in the Research Animal facilities at the University of Wisconsin, which are fully accredited by the American Association for the Accreditation of Laboratory Animal Care. Embryos were staged by both hours post fertilization (hpf) at 28.5°C and by using morphological criteria [71]. To prevent pigment formation, larvae were maintained in E3 containing 0.2 mM N-phenylthiourea (PTU, Sigma Aldrich). For live imaging, 1–3.5 dpf larvae were anesthetized in E3 containing 0.2 mg/mL Tricaine (ethyl 3-aminobenzoate, Sigma Aldrich) and mounted in in 1% low melting point agarose and/or corresponding culture medium. Zebrafish that needed to be euthanized were placed into.05% Tricaine (diluted in E3 water) for 10–15 minutes. After this time the fish were checked to ensure they are not moving or breathing and are then placed into a latex glove for disposal. Alternative way to euthanize: Larval forms between 4 and 10 dpf were maintained in an ice water bath for a minimum of 20 minutes. Death was confirmed when heart and gill movements have ceased.

### Tol2 plasmid injections

Zebrafish embryos were microinjected with a pressure injector with approximately 3 nano-liter volumes at the 1-cell stage. All DNA expression vectors contained the *krt4* promoter for epithelial cell expression [72], [73]. All expression vectors contain minimal Tol2 elements flanking the promoter and gene of interest for efficient integration [74] and an SV40 polyadenylation sequence (Clontech Laboratories, Inc). The following constructs were generated: *krt4-*RFP-HRAS^WT^, *krt4-*RFP-HRAS^V12^, *krt4-*EGFP-L10a and *krt4-*GFP-H2B. Mosaic expression of transgenes was obtained by injecting 3 nano-liter of solution containing 12.5 ng/µL of Tol2 DNA plasmid and 17.5 ng/µL *in vitro* transcribed (Ambion) transposase mRNA into the cytoplasm of one-cell stage embryo.

### Live imaging

For figure 1A, C, D and E, fluorescence images were acquired using Nikon SMZ-1500 zoom microscope equipped with epifluorescence and a CoolSnap ES camera (Roper Scientific, Duluth, GA). For all confocal imaging 1–3.5 dpf larvae were mounted in 1% low melting point agarose and/or corresponding culture medium. For figure 2B–E fluorescence images were acquired using a line scanning confocal microscope (FluoView FV1000, Olympus) using a NA 0.75/20× objective. For figure 3A–B and D–E, Figure 4A–C and F–H, Figure 5A–D and Figure 6C–D fluorescence images were acquired using a spinning disk confocal microscope (Yokogawa CSU-X) with confocal scanhead on a Zeiss Observer Z.1 inverted microscope (NA1.3/63× water immersion objective). Maximum intensity projection images were made using the Zen 2012 (blue edition) software (Carl Zeiss). Neutrophils and macrophages were tracked and analyzed by using plugins MTrackj (3D tracking) and Chemotaxis and Migration tool (ibidi) for ImageJ (NIH, Bethesda, MD). Transformed cell 2D area and cell roundness was measured using the analyze function of ImageJ. Data of time-lapse images represent at least three separate movies. For Figure 2H–I and Figure 4L–M WMISH images were acquired using Nikon SMZ-1500 zoom microscope equipped with a color camera.

### MO mediated Gene knockdown

Morpholino oligonucleotides (Gene Tools) were suspended in distilled water, and stored at room temperature at a concentration of 1 mM. Zebrafish embryos were microinjected with a pressure injector with approximately 3 nano-liter volumes at the 1-cell stage. For *cxcr2* knockdown, a *cxcr2* MO targeting the ATG region (5′- ACTCTGTAGTAGCAGTTTCCATGTT-3′) [18], was used at 100 µM.

For *irf8* knockdown, a previously published splice blocking *irf8* MO [59] (5′-AATGTTTCGCTTACTTTGAAAATGG-3′) [18], was used at 100 µM. For knockdown of exogenous Krt4 driven genes, a *krt4* MO targeting the region directly upstream of the ATG of targets cloned downstream of Krt4 using the BamHI cloning site (*krt*4 MO: 5′-GCTGCTGAGAGACACGCAGAGGGAT-3′) was used at 20 µM (knockdown through 15 hpf). As a control, Gene Tools standard control morpholino (5′- CCTCTTACCTCAGTTACAATTTATA-3′) was used at 100 µM. Gene knockdown was obtained by injecting 3 nano-liter of solution into the cytoplasm of one-cell stage embryo.

### Whole Mount In situ Hybridization

For in whole mount in situ hybridization, Larvae were fixed in 4% paraformaldehyde in PBS and mRNA was labelled by in situ hybridization as previously described [75]. In short, both Dig and fluorescein-labeled antisense probes were hybridized using a 55° hybridization temperature. Purple color was developed with AP-conjugated anti-Dig and BM purple (Roche Applied Science), and red color was developed with AP-conjugated anti-fluorescence and fast red (Roche Applied Science). Reactions were stopped in PBS. Imaging was performed with a Nikon SMZ-1500 stereomicroscope.

The T7 promoter was attached 3′ of the coding sequence of primers to make the DNA templates for the probes. After sequence confirmation of the DNA templates, labelled RNA probes were transcribed with the use of T7 RNA polymerase (Ambion).

Oligo sequences used for PCR were as follows:

Cxcl8 F: 5′-ATGACCAGCAAAATCATTTCAGTGTGTGTTATTG-3′

T7 Cxcl8 R: 5′-TAATACGACTCACTATAGGGAGATCATGGTTTTCTGTTGACAA

TGATCCTATCAATGATC-3′

Mmp9 F: 5′-AAGGAGTTTGACGCCATCAC-3′

T7 Mmp9 R: 5′TAATACGACTCACTATAGGGGAATGGGGTCAATGCAGAAT 3′

Vimentin F: 5′- CTTCAACAATAACCCGCAAA- 3′

T7 Vimentin R: 5′- TAATACGACTCACTATAGGGGGTCAGGTTTGGTCACTTCC -3′

Slug F: 5′-GCATGCCTCGTTCATTCCTA- 3′

T7 Slug R: 5′- TAATACGACTCACTATAGGGGAGGCACTTGTTGAATGCAG -3′

RFP F: 5′-CTTCATGTACGGCAGCAGAA-3′

T7 RFP R: 5′-TAATACGACTCACTATAGGGTGCTAGGGAGGTCGCAGTAT-3′

### Translating ribosome affinity purification (TRAP)

To enrich for transcripts present in RFP-HRas expressing epithelial cells, Tol2 flanked-*krt4*- EGFP-L10a plasmids were co-injected with either Tol2 flanked-Krt4:RFP-HRas^WT^ or Tol2 flanked -Krt4:RFP-HRas^V12^ into one cell zebrafish embryos. We find that co-injection of Tol2 constructs results in their overlapping expression (Figure 2D and E) therefore allowing us to enrich for actively translating RNA from HRas expressing keratinocytes. TRAP was performed as previously published for mRNA purification from zebrafish tissue [53]. In short, 50 3.5 dpf larvae co-expressing L10-EGFP with either HRas^WT^ or HRas^V12^ were homogenized and rabbit anti-GFP antibody (Invitrogen A11122) was used for immunoprecipitation. After immunoprecipitation and high-salt polysome buffer washing steps, RNA was isolated using an RNeasy Mini Kit (Qiagen, Valencia, CA, USA) with in-column DNase digestion. RNA was eluted in 40 µl RNase free water.

### One Step RT PCR

For analysis of tissue specific Cxcr2 expression TRAP was performed on 3.5 dpf transgenic *krt4*-EGFP-L10a and mpx-EGFP-L10a larvae and one-step RT-PCR (QIAGEN) was performed using 2 ul of purified RNA as template. Primers used to amplify ef1α and mpx [76] have been described previously. Primers for cxcr2 were as follows; cxcr2 42 forward, 5′-TCCTTGCCCGGAGACCGTGA -3′; cxcr2 284 reverse, 5′-ATGGTGCCGAACGGCCAGTG-3′. PCR products were analyzed using 1% agarose electrophoresis.

### Quantitative PCR

All quantitative PCR was performed using 2 µl purified TRAP RNA as template. Following RNA isolation, one step qPCR was performed using superscript 3 one step qPCR kit (Invitrogen). All experiments had at least two identical samples and were done in three biological replicates with the reference gene *ef1α* [77] and gene specific primers, which were checked to have produced clean melt curves with one sharp peak indicating specific amplification of the target genes. Fold change was determined using the efficiency-corrected delta comparative quantification method and students t-test (unpaired, two tailed) were performed to determine significance.

Oligo sequences used for qPCR were as follows:

*ef1*α qFw 5′-TGCCTTCGTCCCAATTTCAG- 3′

*ef1α* qRv 5′-TACCCTCCTTGCGCTCAATC- 3′

*vimentin* qFw 5′-GCAGGAGTCTGAGGATTGGT- 3′

*vimentin* qRv 5′-AATCATTGGCCTCCTGTTTG- 3′

*slug* qFw 5′-TTATAGTGAACTGGAGAGTCCAACA- 3′

*slug* qRv 5′-TCCATACTGTTATGGGATTGTACG- 3′

*mmp9* qFw 5′-TGATGTGCTTGGACCACGTAA- 3′

*mmp9* qRv 5′-ACAGGAGCACCTTGCCTTTTC- 3′

### Cell transplants

One cell stage donor embryos were microinjected with approximately 3 nano-liter volumes of injection mixes containing 45 ng/µL in vitro transcribed (Ambion) RFP-HRas^V12^ mRNA mixed with either 100 µM cxcr2 or control MO. During the blastula period, 10–20 cells were transplanted from donor embryos into transgenic Mpx:GFP hosts. Cell transplants resulted in ∼45% success rate of incorporation of RFP labeled donor cells into host embryos. To generate chimeric embryos for TRAP experiments and qPCR analysis one cell stage transgenic *krt4*-EGFP-L10a donor embryos were injected with 3 nano-liter volumes of injection mixes containing 45 ng/µL in vitro transcribed RFP-HRas^V12^ mRNA mixed with either 100 µM cxcr2 MO or control MO and during the blastula period 10–20 cells were transplanted into AB host embryos. Of the chimeric embryos generated ∼60% had RFP expression in epithelial cells in the trunk region and not in the surrounding tissues. These chimeric zebrafish were used for analysis.

### Statistical Analysis

Each *in vivo* experiment was done at least three times. Dot plots contain data from one representative experiment from at least three biological replicates. Each dot is from one foci of HRas labeled cells in the trunk region of an individual embryo and each embryo is represented by one dot. Assuming Gaussian distribution of overall population of values, P values were derived by the following analyses. One-way ANOVA with Dunnett post-test: Figure 3C and F, Figure 4 D–E and I–J, Figure 5E–F, and Figure 6E. Students T-test (unpaired, two tailed) was used in: Figure 1G, Figure 2F–G and J, Figure 4K, Figure 5 G and Figure 6 F. Experimental results were analyzed with Prism version 6 (GraphPad Software) statistical software and standard error is displayed. The resulting P values are included in the figure legends for each experiment.

## Supporting Information

Movie S1 **Live imaging of HRas^WT^ expressing epithelial cells.** Live imaging of epidermal cells in the trunk region of a 3.5 dpf chimeric zebrafish expressing GFP-H2B (green-nucleus) and HRas^WT^ (magenta) in the epidermis. Images were collected at 10 minute intervals for 4 hours. Movie shows the stationary phenotype of HRas^WT^ expressing cells.
(AVI)

Movie S2 **Live imaging of HRas^V12^ expressing epithelial cells.** Live imaging of epidermal cells in the trunk region of a 3.5 dpf chimeric zebrafish expressing GFP-H2B (green-nucleus) and HRas^V12^ (magenta) in the epidermis. Images were collected in 10 minute intervals for 4 hours. Movie shows the dynamic phenotype of HRas^V12^ expressing cells characterized by cell division and active protrusions.
(AVI)

Movie S3 **Neutrophil recruitment to HRas^WT^ expressing cells.** Live imaging of epidermal cells in the trunk region of a 3.5 dpf transgenic *mpx*:GFP (green-neutrophils) chimeric zebrafish expressing HRas^WT^ (magenta). Images were collected for 30 minutes with 2 minute intervals. Movie shows that neutrophils are not recruited to HRas^WT^ expressing cells.
(AVI)

Movie S4 **Neutrophil recruitment to HRas^V12^ expressing cells.** Live imaging of epidermal cells in the trunk region of a 3.5 dpf transgenic *mpx*:GFP (green-neutrophils) chimeric zebrafish expressing HRas^V12^ (magenta). Images were collected for 30 minutes with 2 minute intervals. Movie shows that neutrophils are recruited to HRas^V12^ expressing cells.
(AVI)

Movie S5 **Macrophage recruitment to HRas^WT^ expressing cells.** Live imaging of epidermal cells in the trunk region of a 3.5 dpf transgenic *mpeg*:Dendra (green-macrophages) chimeric zebrafish expressing HRas^WT^ (magenta). Images were collected for 30 minutes with 2 minute intervals. Movie shows that macrophages are not recruited to HRas^WT^ expressing cells.
(AVI)

Movie S6 **Macrophage recruitment to HRas^V12^ expressing cells.** Live imaging of epidermal cells in the trunk region of a 3.5 dpf transgenic *mpeg*:Dendra (green-macrophages) chimeric zebrafish expressing HRas^V12^ (magenta). Images were collected for 30 minutes with 2 minute intervals. Movie shows that macrophages are recruited to HRas^V12^ expressing cells.
(AVI)
